# Effects of an Advocacy Trial on Food Industry Salt Reduction Efforts—An Interim Process Evaluation

**DOI:** 10.3390/nu9101128

**Published:** 2017-10-17

**Authors:** Helen Trevena, Kristina Petersen, Anne Marie Thow, Elizabeth K. Dunford, Jason H. Y. Wu, Bruce Neal

**Affiliations:** 1 Food Policy Division, The George Institute for Global Health, The University of New South Wales, P.O. Box 20, Missenden Road, Sydney NSW 2006, Australia; kpetersen@georgeinstitute.org.au (K.P.); edunford@georgeinstitute.org.au (E.K.D.); Jwu1@georgeinstitute.org.au (J.H.Y.W.); bneal@georgeinstitute.org.au (B.N.); 2 Menzies Centre for Health Policy, School of Public Health, University of Sydney, Sydney NSW 2006, Australia; annemarie.thow@sydney.edu.au; 3 Carolina Population Center, The University of North Carolina at Chapel Hill, Chapel Hill, NC 27516, USA; 4 The School of Public Health, Faculty of Medicine, Epidemiology and Biostatistics, Imperial College of Science, Technology and Medicine, London W2 1PG, UK; 5 Charles Perkins Centre, University of Sydney, Sydney NSW 2006, Australia; 6 The Royal Prince Alfred Hospital, Camperdown, Sydney NSW 2006, Australia

**Keywords:** advocacy, food companies, salt reduction, randomized trial

## Abstract

The decisions made by food companies are a potent factor shaping the nutritional quality of the food supply. A number of non-governmental organizations (NGOs) advocate for corporate action to reduce salt levels in foods, but few data define the effectiveness of advocacy. This present report describes the process evaluation of an advocacy intervention delivered by one Australian NGO directly to food companies to reduce the salt content of processed foods. Food companies were randomly assigned to intervention (*n* = 22) or control (*n* = 23) groups. Intervention group companies were exposed to pre-planned and opportunistic communications, and control companies to background activities. Seven pre-defined interim outcome measures provided an indication of the effect of the intervention and were assessed using intention-to-treat analysis. These were supplemented by qualitative data from nine semi-structured interviews. The mean number of public communications supporting healthy food made by intervention companies was 1.5 versus 1.8 for control companies (*p* = 0.63). Other outcomes, including the mean number of news articles, comments and reports (1.2 vs. 1.4; *p* = 0.72), a published nutrition policy (23% vs. 44%; *p* = 0.21), public commitment to the Australian government’s Food and Health Dialogue (FHD) (41% vs. 61%; *p* = 0.24), evidence of a salt reduction plan (23% vs. 30%; *p* = 0.56), and mean number of communications with the NGO (15 vs. 11; *p* = 0.28) were also not significantly different. Qualitative data indicated the advocacy trial had little effect. The absence of detectable effects of the advocacy intervention on the interim markers indicates there may be no impact of the NGO advocacy trial on the primary outcome of salt reduction in processed foods.

## 1. Introduction

Salt reduction has been a recent focus of efforts to improve the quality of the packaged food supply in a number of countries around the world [1,2]. In Australia, the estimated mean salt intake in adults is 8–10 g/day [3,4]—about double the World Health Organization (WHO) recommended maximum of 5 g/day [5] and the 2017 Suggested Dietary Target of the Australian government also 5 g/day [6]. Raised blood pressure is a leading risk factor for preventable chronic diseases [7] and in all likelihood excess dietary salt is a driver of premature stroke and heart attack in Australia [8]. While there remains some debate about the effects of salt on health [9,10], systematic overviews that summarize the totality of the available data are indicative of harm [11,12]. On this basis, the WHO recommends that all member states seek to achieve a 30% reduction in the mean population intake of salt by 2025 [13].

Processed foods supply 75% of salt in the average Australian diet [14] with salt fulfilling functional, technical, and taste roles [15]. Wide variations in the salt content of comparable foods for sale in Australia highlight differences in both approaches to manufacturing and food company responses to calls for salt reduction from government, industry groups, and civil society [16,17,18,19,20,21]. Established business frameworks [22,23,24,25] are a way of understanding how macro-environmental factors and the competitive landscape can influence company strategy, and by association actions to voluntarily reduce salt, but data describing the impact of public health advocacy as a direct influence are sparse. There is some limited evidence that advocacy targeting public health does have the potential to influence corporate behaviour [26,27,28] but few studies have robustly assessed the effects of advocacy trials on food company actions [29]. To address this, an intervention was designed to assess the effects of advocacy by a non-governmental organization (NGO) to food companies and is described in the study protocol [30]. The intervention sought to promote voluntary salt reduction in Australia and was aligned with the goals of the Australian Food and Health Dialogue (FHD) [31]—a public–private voluntary food reformulation initiative with an aim to reduce salt in processed foods.

As stated in the protocol [30] the primary outcome of the trial is the average salt content of processed foods (mg/100 g) with the goal of making a difference between the randomized groups, whereas the interim outcomes reported here are process-orientated. Process evaluations are useful to provide information on the quality of implementation, and for understanding why an intervention was successful or not [32]. Key considerations to the present study include the quality of the intervention as underpinned by an adapted theory of change and context [32]. While randomization should help to control how context affects the outcome, it is possible that that there were interactions between parts of the intervention and the wider policy and business context at the time [30].

The aim of this process evaluation was to assess the extent to which the advocacy intervention was delivered (the advocacy output) and received by the food companies, and assess the interim outcomes (the advocacy outcomes) while considering the context. 

## 2. Materials and Methods

### 2.1. Advocacy Trial

A total of 45 food companies with Australian-based production, distribution, or marketing operations were included in the trial, and were randomized to receive the advocacy intervention or control. A detailed description of the cluster randomized design and sampling methodology is provided elsewhere [30]. The advocacy intervention was conducted in Australia, and commenced in December 2013. Ethics committee approval was obtained from the University of Sydney Human Research Ethics Committee for the conduct of interviews of the companies with written informed consent obtained from participants. The trial is registered at ClinicalTrials.gov (NCT02373423). 

#### 2.1.1. Included Food Companies

In brief, companies were selected using an established branded food composition database (‘database’) [33] to identify food companies with Australian-based production, distribution, or marketing operations that had 20 or more processed food items recorded in 2011. The major food categories in the database were mapped against the Australian New Zealand Standard Industrial Classification (ANZSIC) to place companies into one or more food processing sectors, for example meat and meat processing, baking, and ‘other processing’—processing not elsewhere classified, such as frozen pre-prepared meals and seasonings [34].

#### 2.1.2. The Intervention

The advocacy intervention ran for 20 months, from December 2013 to July 2015. The intervention was based upon an established theory of change model (COM-B, capability, opportunity, and motivation) [35] adapted to an organizational context. The three aspects of COM-B together with actions commonly used in NGO advocacy informed the design of the intervention (Table 1, Figures S1 and S2). 

The advocacy intervention logic model shown in Figure S1 is described in detail in the study protocol [30] and illustrates the overall design of the advocacy intervention, connections between the theory of change model, and the intended advocacy outcomes. Briefly, the logic model depicts a series of inputs enabling nine intervention functions akin to tools or methods (training, coercion, incentivisiation, persuasion, education, restriction, environmental restructuring, and modelling) which in turn enable advocacy actions. Examples of advocacy actions include framing (where communications purposely promote a public health definition and interpretation of salt reduction to prevent chronic disease [36,37]), promotion of best practice, publication of results of surveillance reports, and letter writing with a call to action. Collectively, the resource inputs, intervention function, and advocacy action formed the advocacy output. The advocacy output was delivered by a small team (<5) of researchers at the NGO. Outputs were directed towards accessible individuals in food companies considered most likely to have a vested interest in improving public health nutrition and innovating for health. This included individuals with nutrition, research, development and senior management roles. The intervention incorporated both programmed and opportunistic elements. 

The first programmed intervention—Intervention 1 (Table 1) was sent to companies with which the NGO was not already engaged and was based upon nutritional data showing where the company ranked in terms of salt content in relevant product categories versus de-identified competitors. The intervention aimed to promote understanding of salt and health, and appeal to values of competition via the provision of competitor data on mean salt content and targeted organizational motivation. The second programmed intervention—Intervention 2—was a letter with a call to action on salt reduction done in collaboration with World Action on Salt and Health (WASH) [38]. The intervention included a letter from the Chairman of WASH citing the contribution of UK food manufacturers to reducing salt in processed foods. The letter was sent to contacts by a researcher together with a call to action asking what food companies could do to support the FHD and emulate the success of their UK counterparts. The third programmed intervention—Intervention 3—was done in the context of the Health Star Rating (HSR) initiative encouraging companies not already working with the NGO to adopt the new front-of-pack labelling system in parallel with salt reduction. Two researchers contacted food companies advising them the NGO had calculated a rating for each of their products recorded in the NGO database, alongside an offer of support in adopting the Health Star Rating front-of-pack labelling system (HSR), announced in June 2014 [39].

Opportunistic elements included providing information, meetings, and requests to meet. One example was a meeting to provide an update on salt reduction progress and exchange data. These elements were informed by information that became available during the course of the intervention period. In particular, the intervention was designed to integrate activity with background initiatives led by the Australian government to promote a healthier processed food supply. In the first instance, these included the FHD [31] and then increasingly, the HSR. 

#### 2.1.3. Control

Food companies assigned to the control had no specific interventions targeting them as part of this study. The control companies were, however, exposed to ongoing background activities. Background activities included publication of the results of surveillance reports using media releases to praise and shame as well as promotion of an understanding of salt reduction and health outcomes. These were delivered as part of the NGO and other non-governmental and governmental initiatives advocating for healthier processed foods and salt reduction. For example, a media release from the NGO widely disseminated the results of a study that evaluated the effects of the FHD targets on the sodium content of three processed food categories [19]. Requests from control group companies made to the NGO team implementing the intervention were responded to and followed up only when not doing so had the potential to bestow an unfair competitive advantage. 

### 2.2. Interim Process Evaluation

Seven interim outcome measures were chosen to examine how the interventions might affect organizational opportunity, motivation, and capability as shown in the logic model. They also provide an indication of the extent to which the intervention was delivered as intended and received by the food companies. The pre-specified interim outcomes in the protocol were:

1. The number of and type of publicly available statements from food companies expressing support for healthier processed foods.
2. The number of and type of publicly available statements from food companies expressing non-support for healthier processed foods.
3. The number of food companies with a nutrition policy published on their website.
4. The level of engagement with the NGO as measured by a count of communications (e.g., email, meetings).
5. The number of companies supporting the use of salt replacers/technologies in food processing to reduce the quantity of sodium required in processing.
6. The number of companies supporting national salt reduction initiatives; and
7. The number of companies providing evidence of planned salt reduction.

In addition, a pre-specified objective of the qualitative assessment was to identify from interview data the organizational capability, opportunity, and motivation to reduce salt across the product portfolio, perceptions of the role of advocacy actions in effecting change, and contextual factors driving nutrition actions.

### 2.3. Data Collection

#### 2.3.1. Seven Interim Outcomes

Data were collected for the period from December 2013 to July 2015 for all interim outcomes. Data for interim Outcome 4 (the level of engagement with the NGO) were collected from a log of all programmed and opportunistic communications that were entered into a standardized Excel spreadsheet. Data for all other interim outcomes were drawn from searches of the Internet. 

The Internet searches were done between September and November 2015 with the goal of identifying (for all companies) the information relevant to the specified interim study outcomes (see Section 2.2). Searches focused on the Australian websites of the companies, but global websites were added as necessary to ensure that all relevant information was captured. Australian Food News [40], Google, and News Bank [41] were the other major sources of information. Search terms included the company trading name (and if appropriate common name or household brand names) together with ‘salt’, ‘sodium’ ‘reduction’ ‘low’, ‘nutrition’, ‘health’ and ‘healthy’. The website of the FHD [31] was checked to identify publicly declared evidence of support for the Australian government initiative to reduce salt. The searches focused on information relating to the period between December 2013 and July 2015, that corresponded to the intervention period. Two authors (H.T. and K.P.) conducted these searches independently. Data were entered into a standardized Excel spreadsheet with data for the first 15 companies collected by both authors and cross-checked to resolve any differences. This ensured alignment of approach for data collection for the remaining 30 companies, which was divided equally between H.T. and K.P. Together, H.T. and K.P. agreed on the final data to report. Information about macro-environmental factors that might have influenced the effects of the intervention on the interim outcomes was recorded as identified during the course of the study, at the interview, and from internet searches.

Macro factors reported in the public domain, and assumed to be influential during the study period, were recorded and filed as they occurred and were used to inform the interviews in year 2.

#### 2.3.2. Interview Data

Semi-structured interviews were conducted with consenting participating food companies in year 1 and year 2 and were done by the same researcher (H.T.) face-to-face or by phone with an average duration of 30 min in year 2. This present study reports interviews in year 2 only. All interviews in year 2 were audio-recorded and transcribed verbatim. For the one interview, where there was a technical issue with the recording, data were transcribed from written notes. The interviews in both years sought to identify views on the status of Australian policy initiatives to reduce salt, other contextual factors that were of concern to the company, and the implications for getting salt reduction onto the company agenda. In year 2, food companies were also asked about the activities of the NGO over the course of the trial to understand how advocacy actions had been received and their impact on company behaviour. 

### 2.4. Analysis

Interim outcome data were assessed using intention to treat analysis, with no imputation for missing data. Statistical significance was defined using a two-sided α = 0.05. Analyses were completed using Stata 13.1 (Stata Corp., College Station, TX, USA). Food company characteristics were summarized for intervention and control groups and explored for chance baseline imbalances. Differences in the interim outcomes between intervention and control groups were assessed by comparing proportions and means depending upon the nature of the variable. We used a negative binomial model for count variables that were over-dispersed, Mann–Whitney U Test for non-parametric data, and the Fisher’s test for categorical data where the numbers were small.

The qualitative analysis of interview data was based upon the data from the semi-structured interviews which were open-coded and organized by two authors (H.T. and A.M.T.) using an inductive/deductive approach and a framing matrix aligned with the logic model. The data were subsequently organized and combined into key themes to report the findings. 

The logic model, which follows an impact pathway approach, was also used to make a summative evaluation of the intervention program to assess whether the interventions were implemented as planned, and to understand the reason for any gaps between intended and actual outcomes.

## 3. Results

### 3.1. Companies Included

**Figure 1:**
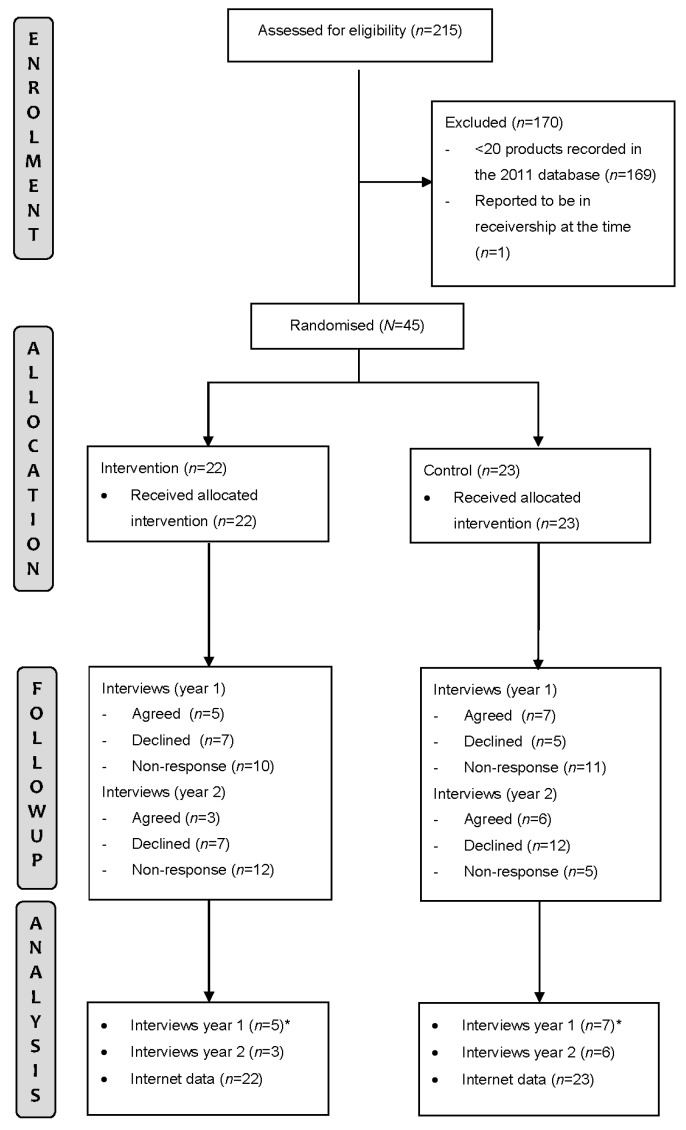
Study flow chart. * Previously analysed and not reported in the present report.

There were 215 potentially eligible companies identified for inclusion with 169 (79%) excluded because they had fewer than 20 products in the database and one (0.5%) because it was reported to be in receivership (Figure 1). Changes to ownership during follow-up occurred for three intervention companies and two control companies. All intervention and control companies were invited to participate in the year 1 and year 2 interviews—there were 12 (five intervention vs. seven control) in year 1, and 9 (three intervention vs. six control) in year 2. Year 1* interviews were previously analysed to inform the trial and are not reported in this present process evaluation report.

Of the 45 companies included, 36 were large (≥200 employees), and 26 were in public ownership. Companies in the intervention and control groups had similar baseline characteristics (*p ≥* 0.05) (Table 2). 

### 3.2. The Intervention Program

There were three rounds of programmed intervention and a series of ongoing opportunistic interventions (sending out of ad hoc information, requests for contact, meetings and information) that were targeted at the intervention group. 

The first programmed intervention (Intervention 1) designed to target organizational motivation achieved a response rate of 31% with half of that being simply an acknowledgement that the information had been received. Intervention 2 targeted both organizational capability and motivation. Nine of 22 companies responded (41%) with six indicating possible action but three being acknowledgement only. Intervention 3 targeted organizational motivation and capability and achieved a response rate of 25%—two responded with acknowledgement and three with an inference of further action.

There were varied additional opportunistic communications targeting organizational opportunity and motivation, for example through the sending out of ad hoc information, requests for contact, meetings, and information exchange between researchers at the NGO. Overall, 16 of the 22 (73%) intervention companies were engaged with a mean of two substantive episodes for each intervention company. A substantive episode typically included ongoing meetings around an issue or the provision of data about the nutritional composition of foods. After all programmed and opportunistic attempts to engage there were still five (23%) intervention companies the NGO was unable to involve during the course of the advocacy trial. Eleven control companies also contacted the NGO spontaneously during the study period, with a mean of 0.7 substantive episodes of interaction each.

Using the logic model (Figure S1) to assess whether the interventions were implemented as planned showed that the trial had not delivered an intervention to support the use of healthier ingredients/technologies, where an interim outcome was the support of low salt alternatives in processing. All other advocacy actions were employed to deliver both opportunistic and programmed communications. Advocacy actions to frame media communications with respect to a consumer right of choice to healthy food, frame the media to praise/shame, and disseminate the research/news policy information to build the knowledge base also formed a large part of the NGO background activities—to which both control and intervention groups were exposed. When assessed by target behaviour, only the interventions targeting support of low salt alternatives (organizational opportunity) and sharing of knowledge on where to go for salt reduction technical advice (organizational capability) were not implemented as planned. 

### 3.3. Interim Outcomes

There were no detectable differences in any of the pre-defined interim outcomes between the intervention and control groups (Table 3). The number of companies that made supportive communications was 15 for intervention companies and 14 for control companies (*p* = 0.63) and the corresponding numbers that were the subject of non-supportive communications were 11 intervention and 12 control companies (*p* = 0.74). Varied supportive communications were delivered as news, comments, and reports, but the mean number made by each company was modest (1.2 intervention vs. 1.4 control). Fewer intervention vs. control companies had a nutrition policy published on their website (23% vs. 46%), although this was not statistically significant (*p* = 0.21). Likewise, the proportion of intervention companies supporting the FHD was 41% vs. 61% of control companies (*p* = 0.24) and the percentages with respect to evidence of a salt reduction plan were 23% vs. 30% (*p* = 0.56). The mean number (range) of communications with the NGO for each intervention company during the study period was 15 (6–53) vs. 11 (5–25) of control companies (*p* = 0.28). 

### 3.4. Qualitative Findings

Nine food companies consented to an interview: three intervention companies and six control companies. The identified drivers of nutrition actions over the 12 months prior to interview were similar for both groups, with no detectable effect of the advocacy intervention. 

The HSR front-of-pack labelling system [39] was identified as the primary driver of company action related to nutrition during the study period. Identified motivating factors for participation in the HSR system were competition, transparency, and congruence with company values. Reasons for not participating were a ‘wait and see’ attitude, insufficient rationale given their product portfolio, and competing business priorities. Implementation of the HSR policy was seen as presenting an opportunity to review the nutritional content of products, including salt content, by several companies. The other government initiative, the FHD, which had a strong focus on salt and reformulation, was noted by all those involved to be dormant and while support for the FHD was evident, none were unduly concerned about the lack of activity. All were following their own nutritional plan—“*we just know what to do in terms of our strategy and we’re following that*” (Company A1).

Consumers were identified as key—“*everything we do is consumer-led*” (Company A2) and many companies identified sugar as the nutrient they believed that consumers were most concerned about. Accordingly, efforts to reduce sugar were a focus compared to (but not necessarily at the expense of) salt reduction. Salt was identified by only one company as having consumer attention and that was for a specific category and consumer demographic. However, most companies indicated that salt was a part of, but not necessarily a priority, of their internal nutritional plans.

Engagements with NGOs in general were clearly separated in terms of those that partnered and those that provoked food companies, with all companies preferring collaboration and partnership. A shared vision and strategy to achieve this were described as prerequisites to maintaining existing working relationships or building future ones—“*with that higher purpose clearly anchoring everything that the NGO (TGI) does I think it would make it much easier for us to connect in and maybe collaborate on trying to deliver that goal*” (Company A3).

Advocacy actions involving the provision of product nutritional data and actions directly related to supporting the implementation of the HSR system resulted in the highest level of engagement. Five companies identified the HSR system implementation process as an opportunity to review the nutritional composition of their products. Two control group companies explicitly described substantial contact over a number of weeks that culminated in a collaborative working relationship as “*much more of a partnership*” (Company A4). The level of engagement was influenced by how accurate the companies perceived the NGO’s nutritional data to be. For example, where the data were perceived to be inaccurate, with a possible threat to their reputation, companies were motivated to engage with the NGO. Another motivation for engaging with the NGO mentioned by two companies was the NGO research expertise in the context of a trusted source of data disseminated through the media, but also in project work. Although all intervention companies mentioned the FHD, HSR, and data, none recalled either of the other two programmed interventions implemented by the NGO.

## 4. Discussion

In this process evaluation we identified no differences between the intervention and control groups in any of the pre-defined interim outcome measures designed to evaluate the interim effect of the advocacy intervention on Australian food companies. This suggests that there may be no impact of the advocacy intervention on the primary downstream outcome of salt reduction, although it is also possible that the chosen interim measures are not indicative of efficacy for that measure.

The advocacy intervention was formulated as a logic model with components targeting organizational opportunity, capability, and motivation based upon the COM-B model for achieving behavioural change [35]. The intervention was designed to encourage collective support for salt reduction and integrate activity with background policy initiatives such as the FHD. The project utilized several intervention functions (enablement, education, modelling, and persuasion) via the provision of data and targeted organizational opportunity, capability, and motivation to reduce salt in foods. It was also intended that the intervention companies would gain knowledge and legitimacy for their nutrition actions as well as a bolstered image of social responsibility [45,46,47].

The possible reasons for failure of the advocacy intervention to impact upon the interim outcomes are multiple. Companies were difficult to engage, with many being minimally or completely non-responsive to the approaches made. Most appeared to have existing corporate positions on salt reduction (and nutrition more broadly) that were difficult to shift with evidence, a referral to a positive overseas experience, or a reference to local government initiatives such as the FHD.

The ability of the NGO to provide food companies nutritional composition data [48] for hundreds of food products over a period of time was a distinguishing feature of the advocacy intervention, and yet this partnership approach still did not elicit a strong response from the intervention companies. This finding could suggest that companies may have already known this information through ongoing competitor tracking [49], did not trust the NGO data, or that salt reduction was not deemed to be of sufficiently high priority [50]. Conversely, referencing the government’s HSR initiative [39] appeared to offer an opportunity because ‘*the marketers now see a front-of-pack benefit in improving nutrition in a product*’ (Company B1), although the potential for salt reduction per se was generally perceived to be curbed with rather few opportunities to differentiate and reformulate in some food categories. A systematic assessment of where each company was on the ‘salt reduction journey’ may have improved the response rate, although the limited number of public documents available to make this assessment, in conjunction with the difficulty of getting corporate participation in interviews, would have made this challenging.

Another possible reason for the absence of a detectable difference in interim outcomes between randomized groups is that the NGO also had multiple interactions with the control group during the study period. This ‘drop in’ likely biased the effects of the advocacy intervention towards the null. While intervention companies were on average targeted with, and exposed to, more intervention, active neglect of the control group would likely have resulted in a greater contrast. Active neglect was not, however, something that was deemed feasible or desirable at the outset of the trial because the NGO was leading NGO salt reduction efforts in Australia. This decision has almost certainly mitigated against a maximally robust test of the hypothesis that advocacy can affect company actions on salt reduction.

This interim process evaluation has shown how the limited resources available to the NGO also adversely impacted the fidelity of the advocacy work with the frequency of programmed interventions being low and tailoring of the advocacy message being limited. Likewise, the capacity of the NGO to conduct and disseminate supporting research, interact with the media and adequately respond to some intervention company requests was challenging. Email as the most convenient and cost-effective mode of delivery was used in the programmed interventions. For each company, a primary point of contact was identified [51] but a more strategic approach targeting multiple individuals with messages tailored to the different and changing roles of the key individuals in each company would almost certainly have achieved better engagement. This might have involved hosting a series of web-based and face-to-face roundtables for senior managers. A failure to appreciate the time and resources required to effectively implement programmed and opportunistic communications was compounded by involvement in background actions such as publishing company comparisons and newsletters to which the control and intervention groups were both exposed. Further, the approach taken was to engage with food companies using tactics that were non-disruptive, are promoted in public health nutrition advocacy [29,52] and aligned to the public-private partnership model of the FHD. The overall advocacy approach tested was one orientated to relationship building to form organizational partnerships—where the level of interaction varied from limited to involved [53].

The inherent conflict of interest between profit from unhealthy food versus health [50], and the over-riding commercial drivers behind the food industry were another issue for which there was no clear solution identified. Public–private partnerships [54,55] like the FHD have the capacity to overcome this challenge, at least in part, if robustly implemented and evaluated [56]. However, while the FHD led with salt reduction when first established, there was almost no activity post-2013, which was the period in which our study was performed. Few companies were willing to step off the sidelines and publicly support a national salt reduction initiative in the absence of leadership from the government. A very similar pattern has been observed with climate change [57] where it is only with the setting of clear long-term goals that real action appears likely to be achieved [58]. The splintered nature of food advocacy organizations and the absence of the coordinated global approach taken by climate advocates provide for a very weak response to a huge and enormously influential food industry [59].

A key strength of the trial was the randomized design selected to overcome the many challenges of quantifying the effects of advocacy in an unbiased way [60,61,62]. While the number of companies included was fairly small, we were able to stratify randomization based on company ownership, size of the company, and industry sector to reduce baseline differences between groups [30]. The adapted theory of change framework we used, although previously untested in this setting, was supported by strong literature, and in conjunction with the logic model provided a highly credible theoretical basis for the intervention program [32,63]. Pre-specification of the main design features in a published protocol protected against post hoc data-driven conclusions. 

However, as we are unaware of process evaluation studies reporting results from comparable advocacy interventions, it is difficult to assess the strengths and weaknesses of this study in relation to others. Evaluations of advocacy to improve the healthfulness of food, particularly in the area of nutrition–obesity prevention, are similarly sparse [64]. Nonetheless, advocacy for salt reduction is widely considered key to changing the government agenda and creating consumer awareness and pressure [65]. Raising consumer awareness of salt and health as part of the UK salt reduction campaign was considered to be fundamental to its success, alongside engaging with the food industry [65,66]. Likewise, we sought to engage with the food industry and the qualitative findings support the need for consumer advocacy given that consumer pressure would almost certainly get the attention of food companies. While background activities included actions to influence public opinion and the government agenda, the design aimed to target food companies directly. Future studies could usefully evaluate the effect of consumer advocacy programs on food company behaviour. 

In terms of weaknesses, and aside from those already identified, the primary shortcomings were the sample size and the limited power to detect plausible small to moderate effects on the interim outcomes. The inclusion of more companies but with fewer products (<20) [30] would have increased the sample size, but the design aimed to include market leaders consistent with the FHD strategy [31]. Access to sales data would determine the likelihood of smaller companies owning a market leading brand and the impact of excluding companies with few products in their portfolio. This process evaluation may have benefited from a comparison of baseline and interim outcomes but it is unlikely this would have substantively changed the overall conclusion given that salt reduction was largely absent from the agenda of the consumer and government at the time the advocacy intervention was initiated. In addition, it is possible that there was insufficient time for the effects of the advocacy intervention to take effect. Finally, the proportion of companies agreeing to the interviews was modest, and it is likely participants in the interviews were different to non-participants—this introduces some uncertainty into the conclusions we drew from the qualitative analysis, although the main messages we highlighted came across so strongly that they seem unlikely to be the result of bias alone. We also sought to enhance objectivity in the qualitative analysis by involving a second researcher (A.M.T) not actively involved in the implementation of the intervention or data collection.

In conclusion, this research suggests no effects of an Australian NGO advocacy program on corporate nutrition actions to reduce salt. The final results will define the ultimate effects on the primary outcome of average salt content in foods, but the present data suggest that this may be a null result. This will have important implications for policy-based initiatives to reduce population salt intake, with the likely conclusions being that either advocacy actions, whether delivered by an NGO, other types of organizations, or coalitions involving a mix of multiple stakeholders, must be much more intensive, or that government-led strategies, alone or in combination with NGO advocacy, will be required to deliver real change. 

## Figures and Tables

**Table 1:** Summary of the interventions and targeted organizational behaviour (capability, opportunity, and motivation).

Advocacy Action	Targeted Behaviour
(a) Opportunistic communications ^1^	Opportunity, motivation
(b) Programmed communication ^1^	Opportunity, motivation
Intervention 1 ^2^—Letter writing with a call to action to promote the understanding of salt and health and appeal to values of competition via the provision of competitor data on mean salt content.	Motivation
Intervention 2 ^3^—Collaboration with WASH. Letter writing with a call to action to promote the success of best practice domestically and overseas.	Capability, motivation
Intervention 3 ^4^—Within the context of Health Star Ratings. Letter writing with a call to action to advise of available (NGO) resources, and disseminate research/news/policy information to build the knowledge base.	Capability, motivation

^1^ includes the sending of ad hoc information, requests for contact, meetings and information; ^2^ A letter targeting companies with whom the NGO had previously been unable to engage; ^3^ A letter targeting all companies; ^4^ A letter targeting companies with whom the NGO was not already sharing nutritional composition data. WASH: World Action on Salt and Health; NGO: non-governmental organization.

**Table 2:** Baseline characteristics of included companies, *N* = 45 (100%).

Baseline Characteristics of Companies	Intervention	Control
	*n* = 22 (%)	*n* = 23 (%)
**Industry sector ^1^**		
Meat-related processing and manufacturing	3 (14)	4 (17)
Dairy, oil and fat-related processing and manufacturing	8 (36)	9 (39)
Cereal, pasta and baking mix manufacturing	9 (41)	8 (35)
Other food product manufacturing	14 (64)	10 (43)
Seafood processing	7 (32)	4 (17)
Fruit and vegetable processing	13 (59)	12 (52)
Bread and bakery manufacturing	8 (36)	6 (26)
Snack-foods and confectionery manufacturing	4 (18)	6 (26)
**Company size**		
Large (≥200 employees)	17 (77)	19 (83)
Small-medium (≤199 employees)	5 (23)	4 (17)
**Company ownership**		
Private	8 (36)	11 (48)
Public	14 (64)	12 (52)
**Participant in Public Health Nutrition initiatives ^2^**		
None	10 (45)	6 (26)
One initiative	3 (14)	6 (26)
Two initiatives	5 (22)	4 (17)
Three initiatives	3 (14)	6 (26)
Four initiatives	1 (5)	1 (5)

^1^ Summary of industry sectors based on the Australian and New Zealand Standard Industrial Classification (ANZSIC) [34]. One or more class descriptions can apply to a single food company. ^2^ Includes: Previous commitment to the Australian Division of World Action on Salt and Health Drop the Salt! Campaign (2007–2012) [42]; Australian Food and Health Dialogue participant [31]; Use of Heart Foundation Tick logo on the front of pack of one or more products 2013 [43]; Member of the Australian Food and Grocery Council Healthier Australia Commitment 2013 [44].

**Table 3:** Comparison of intervention and control group interim outcomes.

Targeted Organizational Change	Interim Outcome	Number of Companies ^1^		Number of Outcomes per Company, Mean (Range) ^1^	
		*n* = 22 (%)	*n* = 23 (%)		
		Intervention	Control	Intervention	Control
*Opportunity, motivation*	**Companies publishing supportive communications:**	**15 (68%)**	**14 (61%)**	**1.5 (0–8)**	**1.8 (0–6)**
	Supportive media releases	2 (9%)	5 (22%)	0.1 (0–1)	0.3 (0–2)
	Supportive other statements	4 (18%)	3 (13%)	0.2 (0–2)	0.2 (0–2)
	Supportive news, comments, and reports	14 (64%)	14 (61%)	1.2 (0–6)	1.4 (0.5)
*Opportunity, motivation*	**Total number of unsupportive communications:**	**11 (50%)**	**12 (52%)**	**1 (0–6)**	**1 (0–4)**
	Unsupportive media releases	0	0	0	0
	Unsupportive other statements	1 (5%)	1 (4%)	<0.01 (0–1)	<0.01 (0–1)
	Unsupportive news, comments, and reports	11 (50%)	11 (48%)	1 (0–5)	0.9 (0–4)
*Opportunity, motivation, capability*	**Total number of NGO communications:**	**22 (100%)**	**23 (100%)**	**15 (6–53)**	**11 (5–25)**
	Update meetings	9 (41%)	4 (17%)	0.8 (0–5)	0.3 (0–3)
	Data exchange	3 (14%)	5 (22%)	0.1 (1–0)	0.4 (1–3)
	Events	6 (27%)	4 (17%)	0.8 (1–5)	0.2 (1–2)
	Project/ongoing work	5 (23%)	3 (13%)	0.3 (1–2)	0.2 (1–2)
*Motivation*	**Companies with a nutrition policy**	**5 (23%)**	**10 (44%)**	**-**	**-**
*Motivation*	**Companies supporting the Australian Food and Health Dialogue**	**9 (41%)**	**14 (61%)**	**-**	**-**
*Motivation, capability*	**Companies providing evidence of planned salt reduction**	**5 (23%)**	**7 (30%)**	**-**	**-**
*Motivation, capability*	**Companies using salt replacer technology**	**-**	**-**	**-**	**-**

^1^ There were no statistically significant differences between the outcome measures (*p* > 0.05 for all pairwise comparisons).

## Supplementary Materials

The following are available online at www.mdpi.com/2072-6643/9/10/1128/s1, Figure S1: Advocacy intervention logic model, Figure S2: Change model.
