# Supplementary material for: Effects of an Advocacy Trial on Food Industry Salt Reduction Efforts—An Interim Process Evaluation
Source: Nutrients. 2017 Oct 17;9(10):1128. doi: 10.3390/nu9101128 (PMC5691744; doi:10.3390/nu9101128)
Supplement: Supplementary file 1 [file nutrients-09-01128-s001.zip › Zip files for Nutrients/Figure S1_Intervention program logic model_140717.pdf]

Figure S1 Intervention program logic model

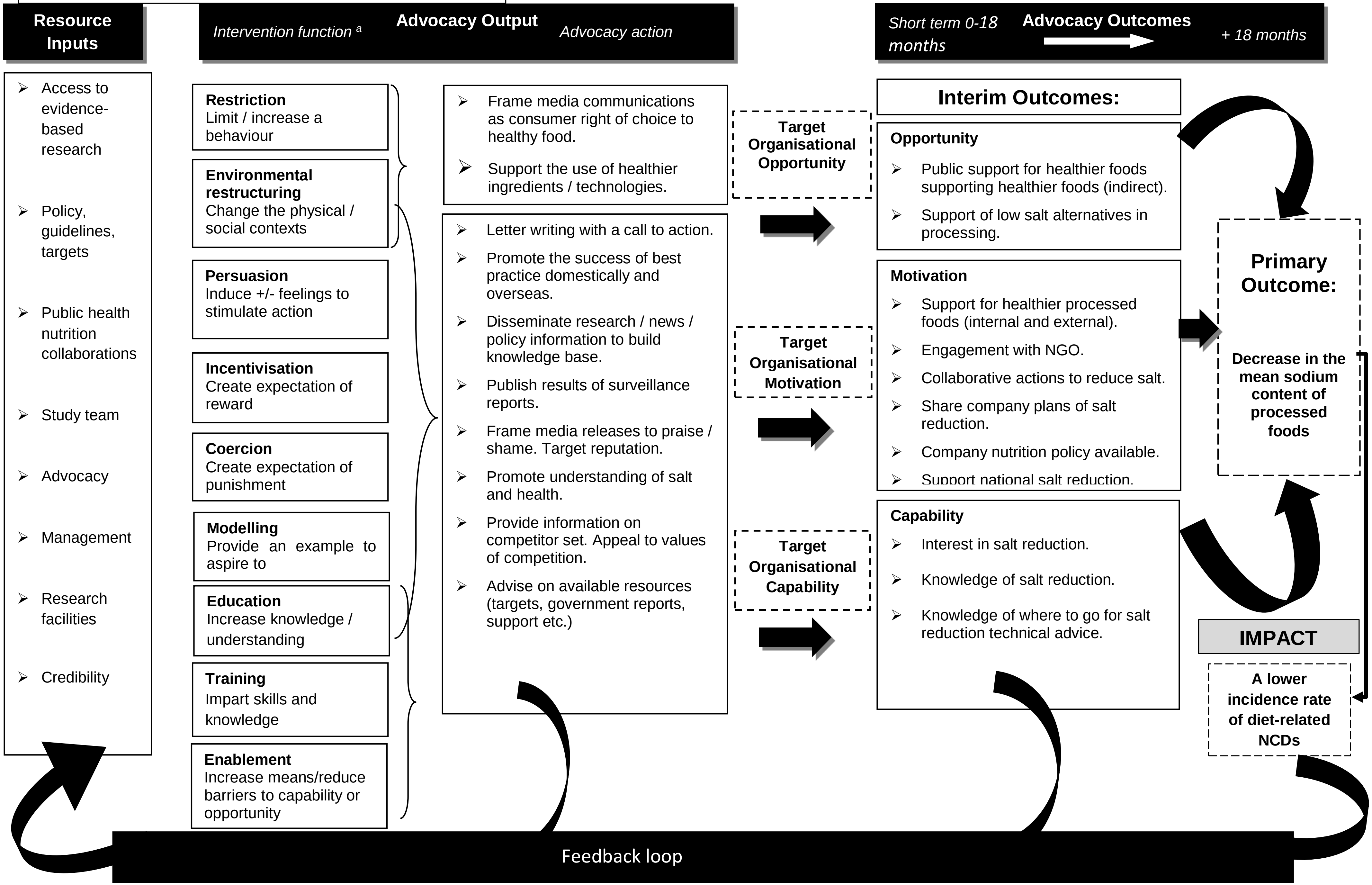


[a]Adapted from Michie S, van Stralen M, West R. The Behaviour change wheel a new method for characterising and designing behaviour change interventions. Implement Sci. 2011,6:42
