# Supplementary material for: Effects of an Advocacy Trial on Food Industry Salt Reduction Efforts—An Interim Process Evaluation
Source: Nutrients. 2017 Oct 17;9(10):1128. doi: 10.3390/nu9101128 (PMC5691744; doi:10.3390/nu9101128)
Supplement: Supplementary file 1 [file nutrients-09-01128-s001.zip › Zip files for Nutrients/Figure S2 Change model 140717.pdf]

Figure S2 Change model: the original behavioural model by Michie et al. [1] of capability, motivation, opportunity and behaviour (COMB-B) is shown in grey. The organisational dimensions in the context of a healthier processed food supply are shown in the boxes with a dotted line.

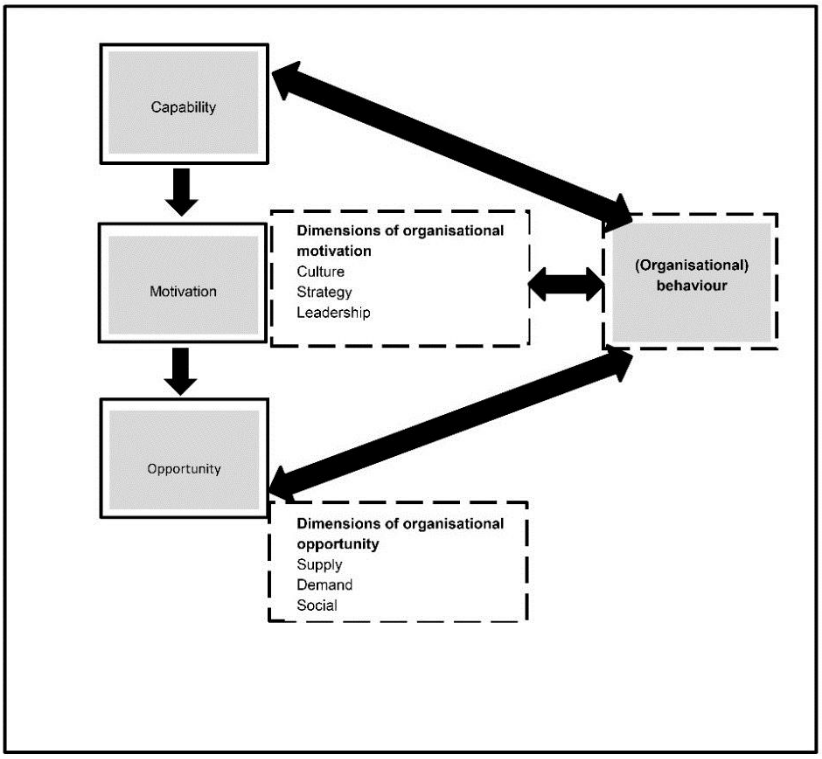

O*rganisational capability* is viewed as the technical, managerial, and process competencies necessary to improve the nutritional profile of processed foods [2]. *Organisational opportunity* comprises the physical supply and demand considerations where 'supply' is a set of up-stream activities (procurement and the availability and access to less salty ingredients) and 'demand' a set of down-stream activities (distribution channels, consumer preferences) and social opportunity [1] defined as the opportunity to contribute to the prevailing discourse and determined by organisational culture [3] (communications strategy, and framing). *Organisational motivation* is considered to be the mix of planned and unplanned events prompting an organisation to choose certain behaviours which may be planned positions on nutrition integral to the company culture or emergent positions responding to a threat to reputation or other issues [4].

References

1. Michie S, van Stralen M, West R. The behaviour change wheel: a new method for characterising and designing behaviour change interventions. Implement Sci. 2011; 6:42:1-11
2. Wong WP, Tseng M-L, Tan KH. A business process management capabilities perspective on organisation performance. Total Qual Manag Bus. 2013;25:602-17.
3. Hendriks A-M, Jansen M, Gubbels J, De Vries N, Paulussen T, Kremers S. Proposing a conceptual framework for integrated local public health policy, applied to childhood obesity - the behavior change ball. Implemt Sci. 2013;8:1-16.
4. Smith M, Busi M, Ball P, Van Der Meer R. Factors influencing an organisation's ability to manage innovation: a structured literature review and conceptual model. International Journal of Innovation Management (IJIM). 2008;12:655-76.
